# Supplementary material for: Application of a time-series deep learning model to predict cardiac dysrhythmias in electronic health records
Source: PLoS One. 2021 Sep 13;16(9):e0239007. doi: 10.1371/journal.pone.0239007 (PMC8437288; doi:10.1371/journal.pone.0239007)
Supplement: Attachment — Submitted filename: Response to reviewers.docx [file pone.0239007.s001.docx]

**Response to reviewer comments**

We would like to thank the editor and the reviewer for the great and helpful comments. Please find our point-by-point response to the reviews below in bold. We hope you find the revised manuscript suitable for publication.

**Journal Requirements:**

When submitting your revision, we need you to address these additional requirements.

1. Please ensure that your manuscript meets PLOS ONE's style requirements, including those for file naming. The PLOS ONE style templates can be found at

<https://journals.plos.org/plosone/s/file?id=wjVg/PLOSOne_formatting_sample_main_body.pdf> and <https://journals.plos.org/plosone/s/file?id=ba62/PLOSOne_formatting_sample_title_authors_affiliations.pdf>

**Thank you for the instructions, we have modified the manuscript according to the instructions.**

2. Our internal editors have evaluated your manuscript and determined that it is within the scope of our 'Primary and Secondary Prevention of Cardiovascular Disease' Call for Papers. This collection of papers is headed by a team of Guest Editors for PLOS ONE and will encompass a diverse range of research articles. Additional information can be found on our announcement page: (<https://collections.plos.org/s/prevention-cardiovascular>). If you would like your manuscript to be considered for this collection, please let us know in your cover letter and we will ensure that your paper is treated as if you were responding to this call. If you would prefer to remove your manuscript from collection consideration, please specify this in the cover letter.

**Thank you for considering our manuscript within the scope of the call for papers. We would like our manuscript to be considered for the suggested collection and we have modified our cover letter accordingly.**

3. In ethics statement in the manuscript and in the online submission form, please provide additional information about the patient records used in your retrospective study. Specifically, please ensure that you have discussed whether all data were fully anonymized before you accessed them and/or whether the IRB or ethics committee waived the requirement for informed consent. If patients provided informed written consent to have data from their medical records used in research, please include this information.

**Thank you. We have added the detail, “All the data were fully anonymized before we accessed them.”**

4. We note that you have indicated that data from this study are available upon request. PLOS only allows data to be available upon request if there are legal or ethical restrictions on sharing data publicly. For information on unacceptable data access restrictions, please see

<http://journals.plos.org/plosone/s/data-availability#loc-unacceptable-data-access-restrictions>.

**Thank you. In our Data Availability statement, we stated that we will be able to share the Python code related to the analyses at the time of acceptance of the manuscript. As the data are owned by a third party, we do not have permission to share the data. Requesting access to The Guideline Advantage (TGA) data must be done by contacting the American Heart Association via email:** [**qualityresearch@heart.org**](mailto:qualityresearch@heart.org)**.**

**Reviewers' comments:**

Reviewer's Responses to Questions

Comments to the Author

1. Is the manuscript technically sound, and do the data support the conclusions?

The manuscript must describe a technically sound piece of scientific research with data that supports the conclusions. Experiments must have been conducted rigorously, with appropriate controls, replication, and sample sizes. The conclusions must be drawn appropriately based on the data presented.

Reviewer #1: No

**Thank you for your helpful review. We have added more details about the technical description and conducted more rigorous experiments according to the reviewers’ comments and suggestions.**

2. Has the statistical analysis been performed appropriately and rigorously?

Reviewer #1: No

**We appreciate the opportunity to improve upon our previous statistical analyses, and have conducted our experiments more rigorously according to the reviewers’ comments and suggestions.**

3. Have the authors made all data underlying the findings in their manuscript fully available?

The [PLOS Data policy](http://www.plosone.org/static/policies.action#sharing) requires authors to make all data underlying the findings described in their manuscript fully available without restriction, with rare exception (please refer to the Data Availability Statement in the manuscript PDF file). The data should be provided as part of the manuscript or its supporting information, or deposited to a public repository. For example, in addition to summary statistics, the data points behind means, medians and variance measures should be available. If there are restrictions on publicly sharing data—e.g. participant privacy or use of data from a third party—those must be specified.

Reviewer #1: No

**Thank you for calling our attention to the details of this policy. Please find the data availability statement in our manuscript as follows.**

**“Data availability statement**

**The data are owned by a third party and the authors do not have permission to share the data. Requesting access to The Guideline Advantage (TGA) data must be done by contacting the American Heart Association via email:** [**qualityresearch@heart.org**](mailto:qualityresearch@heart.org)**. The study team will be able to share the code related to the analyses at the time of acceptance of the manuscript.”**
4. Is the manuscript presented in an intelligible fashion and written in standard English?

PLOS ONE does not copyedit accepted manuscripts, so the language in submitted articles must be clear, correct, and unambiguous. Any typographical or grammatical errors should be corrected at revision, so please note any specific errors here.

Reviewer #1: Yes

5. Review Comments to the Author

Please use the space provided to explain your answers to the questions above. You may also include additional comments for the author, including concerns about dual publication, research ethics, or publication ethics. (Please upload your review as an attachment if it exceeds 20,000 characters)

**Reviewer #1:** In their manuscript titled "Application of a time-series deep learning model to predict cardiac dysrhythmias in electronic health records", Guo et al use the Electronic healthcare records (EHR) data available in The Guideline Advantage (TGA) and deep learning methodologies to make predictions on cardiac dysrythimas (CD) based on cardiovascular health (CVH) measures. They propose that LSTM performs better than LR, RF and NB and identify blood pressure and BMI as the most influential features.

Extracting time series information based on EHR data is a topic of high interest in the community and the question the authors pose, namely "can we predict CD based on EHR data using machine learning methodologies?" is very timely because the quality and quantity of available data is increasing and machine learning techniques have become not only more powerful but also more interpretable and robust. The authors states that the paper is the first time that DL algorithms have been applied to predict CD using time series EHR data. However, the manuscript has two major shortcomings; the methodology is not described in detail to be reproducible and the time dependent information does not reflect real time.

**Thank you for these helpful comments. We have conducted more analyses and revised the manuscript accordingly. We made detailed changes, including: (1) adding more descriptive language to make the methodology more clear; (2) uploading the code to Github; (3) conducting and comparing the prediction analysis using categorical values of events (without Word2Vec) and also feed-forward neural networks; (4) conducting all prediction analyses using 5-fold cross validation; (5) adding more error metrics for evaluating model performance; and (6) conducting statistical significance analyses for comparing metrics for different models.**

Time dependence: DL has been used for different classification tasks on EHR and CD prediction using different types of datasets (Solares 2020, Xia 2018, Shashikumar 2017). This study builds on literature by applying existing methods to a previously studied dataset. The authors state that this is the first that DL is applied to predict CD using time series EHR data. Indeed, if time dependence is incorporated into the predictive model, this is very interesting. However, it is not clear how time is included in the representations. The authors also mention this in the Limitations section. The events are represented by 311 time steps. Are two events separated by two days treated the same way as two events separated by two months? The authors state they use padding to create events so that they can have a 311D vector. How is this padding performed? If dynamic data is the major contribution of the paper, more information about the treatment of time should be included. Furthermore, it is not clear how blood pressure and BMI are the most influential features are impacted by time dependence.

**Thank you for the great points. We have modified the manuscript according to these suggestions. Specifically,**

**(1) We have added citations for the three references:**

***a. Deep learning for electronic health records: A comparative review of multiple deep neural architectures;***

***b. Opportunities and challenges in developing deep learning models using electronic health records data: a systematic review; and***

***c. A deep learning approach to monitoring and detecting atrial fibrillation using wearable technology.***

**(2) Time dependence has been incorporated in the predictive models. In the methods section, we stated that “We also added time information for all measurements as time steps. Each feature was associated with a time point which was calculated by the difference in days between the corresponding visit time and the latest measurement time. For example, if the most recent visit date was February 11, 2019, and the measurement was conducted on January 11, 2019, then the time point value is 31.”**

**(3) For the padding to the same length as 311 (the maximum number of measures), we padded patients with virtual events (which were represented as 0) before the earliest events if patients had fewer than 311 measures, like [0_1_, … 0_k_, event_1, event_(311-k)]. This padding method was inspired by the technique employed by Rajkomar et. al (2018).**

Word2Vec: The study first constructs categorical features to represent patients and then learns continuous vectors usingWord2Vec algorithm. The authors can compare Word2Vec with categorical representations to show the value added by distributed representations. Besides, the construction of categorical vectors and the association of vectors with time can be detailed further, since these are key to the representations.

**Thank you for the great point. We have added this analysis by comparing the effects of categorical features to continuous vectors generated by the Word2Vec algorithm. For the categorical analysis, we used categorical features concatenated with time points (e.g., difference in days between the corresponding visit time and the latest measurement time).**

**We have added the description of comparing continuous with categorical representations as follows.**

**“To investigate the effects by continuous vectors obtained from the Word2Vec algorithm, we conducted the same analysis of predictions by using categorical variables. These categorical variables were sorted in a time order, and each categorical event was concatenated with the same time points as another feature (e.g., difference in days between the corresponding visit time and the latest measurement time), which were the inputs of the models of LSTM, DNN, RF, LR, and NB. We did the same padding approach as above for patients had less events than 311.”**

LSTM: LSTM is adopted as a neural time-series model to identify CD patients and it is compared with three benchmark models: Naive Bayes, Logistic Regression and Random Forest. Given that these models are neither designed to classify time-series nor as complex as a neural network, other models, such as non-neural time-series models and feed-forward neural networks, can be added as benchmarks to reveal the benefits of LSTM further. This would also uncover if the performance gain is due to time-series representation or complexity of neural models.

**Thank you for the great suggestions. We have added feed-forward neural networks to the analyses, and compared them with LSTM. The results are in Figure 4 and Table 4.**

**For the classical time-series models (i.e., non-neural time series models), we were unable to identify the appropriate model considering that the label variable (i.e., presence of CD or not) is not a time-series random variable.**

Causation: The study concludes that “BMI and blood pressure could lower the chance of developing CD” based on the fact that the removal of these features affected the prediction performance the most. This association can be analyzed in more detail, since these features are first embedded using Word2Vec and, thus, not directly observed by the prediction model. Moreover, the causation can be shown explicitly, since the performance drop might not necessarily indicate a causation but might simply mean that these features helped model to discriminate CD patients from the healthy group the most.

**Thank you for the great comments. We have added more details to the manuscript in this regard. Specifically, for each feature, we first manually set it as a constant (not informative for the prediction), then tested the prediction performance of trained models using the manually-changed features to evaluate the discriminative importance of the given feature.**

**We have modified the language to indicate that these features helped the model to discriminate CD patients from the healthy group the most, instead of saying these features were the most important features to lower the chance of developing CD.**

DL model: The code is not yet available. The authors have not included important details about the algorithms used. For example, how do the authors test for overfit? Is 5-fold cross validation performed? What is the error in their performance metrics? What is the statistical significance of their model comparison metrics?

**Thank you for requesting we provide the code. The Python code is now available in Github:**

[**https://github.com/aixiaguo/CD_prediction/blob/master/CD.py**](https://github.com/aixiaguo/CD_prediction/blob/master/CD.py)

**We have added 5-fold cross validations for all models. We corrected an error in the data-preprocessing, and we re-ran all the analyses.**

**We have also added more performance metrics, e.g., overall accuracy, precision, recall, f1-score and specificity. The results are shown in the Table 4.**

**We have also compared the statistical significance for metrics from different models by one-tailed t-test (Table 5). For example, there were 5 values of accuracy from the 5-fold cross validation for LSTM model and DNN model. We performed a one-tailed t-test on these values of accuracy to determine the statistical significance. The p-values in Table 5 show that all of the LSTM model performance metrics were significantly higher than other models.**

**Table 4. Model performance by metrics of 5-fold cross-validation mean (std).**

| **Cases** | **Models** | **Accuracy** | **Precision** | **Recall** | **f1** | **Specificity** |
| --- | --- | --- | --- | --- | --- | --- |
| **Case:**  **Inputs with vectors by Word2Vec** | LSTM | 0.9 (0.01) | 0.9 (0.02) | 0.89 (0.02) | 0.89 (0.01) | 0.91 (0.03) |
|  | DNN | 0.8 (0.03) | 0.78 (0.04) | 0.82 (0.05) | 0.8 (0.03) | 0.79 (0.05) |
|  | RF | 0.74 (0.01) | 0.75 (0.01) | 0.7 (0.04) | 0.73 (0.02) | 0.79 (0.03) |
|  | LR | 0.65 (0.01) | 0.65 (0.01) | 0.58 (0.02) | 0.61 (0.01) | 0.71 (0.02) |
|  | NB | 0.52 (0.0) | 0.58 (0.13) | 0.01 (0.01) | 0.02 (0.02) | 0.99 (0.01) |
| **Case:**  **Inputs without Word2Vec** | LSTM | 0.84 (0.01) | 0.84 (0.01) | 0.82 (0.01) | 0.83 (0.01) | 0.86 (0.01) |
|  | DNN | 0.72 (0.01) | 0.71 (0.01) | 0.7 (0.01) | 0.71 (0.01) | 0.74 (0.02) |
|  | RF | 0.68 (0.01) | 0.74 (0.01) | 0.51 (0.04) | 0.6 (0.03) | 0.84 (0.01) |
|  | LR | 0.56 (0.01) | 0.57 (0.02) | 0.37 (0.02) | 0.45 (0.02) | 0.74 (0.02) |
|  | NB | 0.52 (0.0) | 0.39 (0.37) | 0.0 (0.0) | 0.0 (0.0) | 1.0 (0.0) |

**The calculation of metrics was based on the following formulas.**

***Accuracy = (TP + TN)/(TP + TN + FP + FN)***

***Sensitivity = TP/(TP + FN)***

***Specificity = TN/(TN + FP)***

***Precision = TP/(TP + FP)***

***F1-score = 2TP/(2TP + FP + FN)***

**Where *TP* is true positive, *TN* is true negative, *FP* is false positive and *FN* is false negative.**

**Table 5. Statistical significance of model comparison metrics.**

| **Cases** | **Metrics** | **(LSTM, DNN)** | **(LSTM, RF)** | **(LSTM, LR)** | **(LSTM, NB)** |
| --- | --- | --- | --- | --- | --- |
| **Case:**  **Inputs with vectors by Word2Vec** | **AUC** | 5.5*10^-4^ | 6.8*10^-9^ | 5.6*10^-11^ | 4.8*10^-11^ |
|  | **Accuracy** | 2.6*10^-4^ | 1.5*10^-8^ | 4.6*10^-10^ | 5.0*10^-12^ |
|  | **Precision** | 2.6*10^-4^ | 9.2*10^-7^ | 2.6*10^-8^ | 6.5*10^-4^ |
|  | **Recall** | 0.01 | 1.5*10^-5^ | 6.1*10^-9^ | 3.1*10^-14^ |
|  | **F1-score** | 3.0*10^-4^ | 1.2*10^-7^ | 3.9*10^-10^ | 1.8*10^-13^ |
|  | **Specificity** | 0.001 | 7.1*10^-5^ | 1.4*10^-6^ | 1.3*10^-4^ |
| **Case:**  **Inputs without Word2Vec** | **AUC** | 5.8*10^-9^ | 1.3*10^-7^ | 3.6*10^-12^ | 6.2*10^-10^ |
|  | **Accuracy** | 5.1*10^-8^ | 9.5*10^-9^ | 2.8*10^-11^ | 8.1*10^-13^ |
|  | **Precision** | 2.5*10^-7^ | 6.5*10^-7^ | 3.0*10^-9^ | 0.02 |
|  | **Recall** | 2.2*10^-7^ | 9.8*10^-8^ | 1.2*10^-10^ | 3.7*10^-15^ |
|  | **F1-score** | 2.7*10^-8^ | 9.8*10^-8^ | 4.0*10^-11^ | 2.71*10^-16^ |
|  | **Specificity** | 2.0*10^-6^ | 0.03 | 1.7*10^-5^ | 3.9*10^-8^ |

The manuscript is well organized and easy to follow but more details about the methodology are needed and the conclusions are not supported by the experiments.

**Thank you so much again for all the great comments. We have revised the methodology and conclusions suggested as above accordingly. We hope you find the revised manuscript suitable for publication.**

a	b

**Figure 4. CD prediction performance by area under the curve (AUC) for LSTM, DNN, RF, LR, and NB models.** LSTM – long short-term memory; DNN – deep neural networks; RF – random forest; NB – naïve Bayes. (a) using Word2Vec embedding vectors as inputs for models; (b) not using Word2Vec embedding vectors as inputs for models.

**Fig 5**. **Feature discriminative importance evaluated using the LSTM model.**
