# Supplementary material for: Application of a time-series deep learning model to predict cardiac dysrhythmias in electronic health records
Source: PLoS One. 2021 Sep 13;16(9):e0239007. doi: 10.1371/journal.pone.0239007 (PMC8437288; doi:10.1371/journal.pone.0239007)
Supplement: Attachment — Submitted filename: Response to reviewers.docx [file pone.0239007.s002.docx]

**Response to reviewer comments**

*We would like to thank the reviewers for their informed, thoughtful, and helpful comments. Please find our responses to the reviews below in italics. We believe that the manuscript has been significantly improved by our responses to the reviewers and hope that you will find it suitable for publication in the PLOS ONE.*

**Reviewers' comments:**

Reviewer's Responses to Questions

**Comments to the Author**
1. If the authors have adequately addressed your comments raised in a previous round of review and you feel that this manuscript is now acceptable for publication, you may indicate that here to bypass the “Comments to the Author” section, enter your conflict of interest statement in the “Confidential to Editor” section, and submit your "Accept" recommendation.

Reviewer #1: All comments have been addressed

2. Is the manuscript technically sound, and do the data support the conclusions?

The manuscript must describe a technically sound piece of scientific research with data that supports the conclusions. Experiments must have been conducted rigorously, with appropriate controls, replication, and sample sizes. The conclusions must be drawn appropriately based on the data presented.

Reviewer #1: Yes

3. Has the statistical analysis been performed appropriately and rigorously?

Reviewer #1: Yes

4. Have the authors made all data underlying the findings in their manuscript fully available?

The [PLOS Data policy](http://www.plosone.org/static/policies.action#sharing) requires authors to make all data underlying the findings described in their manuscript fully available without restriction, with rare exception (please refer to the Data Availability Statement in the manuscript PDF file). The data should be provided as part of the manuscript or its supporting information, or deposited to a public repository. For example, in addition to summary statistics, the data points behind means, medians and variance measures should be available. If there are restrictions on publicly sharing data—e.g. participant privacy or use of data from a third party—those must be specified.

Reviewer #1: Yes

5. Is the manuscript presented in an intelligible fashion and written in standard English?

PLOS ONE does not copyedit accepted manuscripts, so the language in submitted articles must be clear, correct, and unambiguous. Any typographical or grammatical errors should be corrected at revision, so please note any specific errors here.

Reviewer #1: Yes

6. Review Comments to the Author

Please use the space provided to explain your answers to the questions above. You may also include additional comments for the author, including concerns about dual publication, research ethics, or publication ethics. (Please upload your review as an attachment if it exceeds 20,000 characters)

**Reviewer #1:**

**The authors have addressed the concerns. The authors can include some references to time aware LSTM for future work (eg DOI: 10.1145/3097983.3097997).**

*Thank you. Our apologies for not adding these references in the original manuscript. We have added the following references and also added to the discussion section as the future work.*

*1.Baytas IM, Xiao C, Zhang X, Wang F, Jain AK, Zhou J. Patient subtyping via time-aware LSTM networks. In: Proceedings of the ACM SIGKDD International Conference on Knowledge Discovery and Data Mining. 2017.*

*2.Zhang Y, Yang X, Ivy J, Chi M. Attain: Attention-based time-aware LSTM networks for disease progression modeling. IJCAI International Joint Conference on Artificial Intelligence. 2019. doi:10.24963/ijcai.2019/607.*

**Dear reviewer,**

We made an error in data pre-processing during the last revision process. We have since found the error, corrected it, and reproduced the related results (specifically, Figures 4 & 5, and Tables 4 & 5). All of the original conclusions and patterns still hold and the values (Figures 4 & 5) of the results are similar to the initial submission, prior to the submitted revision. Tables 4 & 5 were newly added during the last revision by suggestions from reviewers.

To provide additional context, the final values of the results (Figures 4 & 5, and Tables 4 & 5) we now include in the manuscript for your kind consideration.

We apologize for any inconvenience this may cause and are grateful for your time and expertise in assessing our manuscript. We hope you find the revised manuscript suitable for publication; in the meantime, please let us know if you have any questions or concerns.

Sincerely,

Randi Foraker, PhD, MA, FAHA

Associate Professor of Medicine

Washington university in St Louis
